# Spermidine Improves Freezing Tolerance by Regulating H_2_O_2_ in *Brassica napus* L.

**DOI:** 10.3390/antiox13091032

**Published:** 2024-08-26

**Authors:** Shun Li, Yan Liu, Yu Kang, Wei Liu, Weiping Wang, Zhonghua Wang, Xiaoyan Xia, Xiaoyu Chen, Chen Wang, Xin He

**Affiliations:** 1 College of Agronomy, Hunan Agricultural University, Changsha 410128, China; lishun@stu.hunau.edu.cn (S.L.); liuyan@stu.hunau.edu.cn (Y.L.); kangyu1226@stu.hunau.edu.cn (Y.K.); liuwei2020@stu.hunau.edu.cn (W.L.); wwp@stu.hunau.edu.cn (W.W.); wangzhonghua@stu.hunau.edu.cn (Z.W.); xiaxiaoyan@stu.hunau.edu.cn (X.X.); chenxiaoyu339@stu.hunau.edu.cn (X.C.); wangchen8548@stu.hunau.edu.cn (C.W.); 2 Yuelushan Laboratory, Changsha 410128, China

**Keywords:** *Brassica napus* L., polyamines, freezing stress, H_2_O_2_

## Abstract

Low temperature is a common abiotic stress that causes significant damage to crop production. Polyamines (PAs) are a class of aliphatic amine compounds that serve as regulatory molecules involved in plant growth, development, and response to abiotic and biotic stresses. In this study, we found that the exogenous application of two concentrations of spermidine (Spd) significantly enhanced the freezing tolerance of three differently matured rapeseed (*Brassica napus* L.) varieties, as manifested by higher survival rates, lower freezing injury indexes, and reduced H_2_O_2_ content. RNA-seq and qRT-PCR analyses showed that Spd enhanced the freezing tolerance of rapeseed by regulating genes related to the PA metabolic pathway and antioxidant mechanism, and generally inhibited the expression of genes related to the JA signaling pathway. This study provides a reference basis for understanding the functionality and molecular mechanisms of polyamines in the response of rapeseed to freezing stress.

## 1. Introduction

Rapeseed (*Brassica napus* L.) is one of the most important oil crops in the world. However, the growth and development of rapeseed are severely impacted by environmental conditions. In recent years, due to global climate change, extreme weather events have become more frequent, leading to an increasing risk of crop yield loss due to low-temperature stress.

Polyamines (PAs) are a kind of small molecular aliphatic amine compound containing two or more amino groups. According to the number of amino groups and molecular structures, PAs can be divided into putrescine (Put), spermidine (Spd), spermine (Spm), thermospermine (Tspm), cadaverine (Cad), etc. Among them, Put, Spd, Spm, and Tspm are regarded as the major PAs in plant organisms and are widely present in various plants [1]. Based on their forms of existence, plant PAs mainly exist in three forms: free PAs (F-PAs), conjugated covalent PAs (CC-PAs), and non-conjugated covalent PAs (NCC-PAs) [2]. In higher plants, PAs mainly exist in the form of free PAs and can form conjugated polyamines (PSCC-PAs) by covalently binding with small molecules [3,4], participating in plant morphogenesis and responses to stresses [5,6,7].

In higher plants, the endogenous level of PAs is affected by the developmental stage and external environment, and its level is strictly controlled within a certain range through metabolic and catabolic pathways, which in turn regulate the growth, development, and stress response in plants [6,8]. In general, polyamines directly participate in translation regulation or indirectly integrate into signaling pathways by generating ROS signaling molecules via catabolic pathways [6]. PAs are oxidized by a diverse array of enzymes, including copper-containing amine oxidases (CuAOs) and flavin adenine dinucleotide (FAD)-dependent polyamine oxidases (PAOs). CuAOs primarily catalyze the oxidation of Put and Cad, with less efficiency in oxidizing the primary amino groups of Spd and Spm. PAO is the key enzyme that catalyzes the oxidation and mutual transformation of polyamines. This oxidation process results in the production of ammonia, hydrogen peroxide (H_2_O_2_), and an aldehyde derivative. The products, such as H_2_O_2_, produced in this process, as ROS signals, mediate various aspects of plant growth and development (such as cell differentiation, seed germination, leaf senescence, fruit ripening, etc.) and responses to adverse stresses (high temperature, low temperature, salt stress, etc.) [9,10,11,12].

Previous studies on various plant species have shown that PA accumulation occurs in response to several adverse environmental conditions, including extreme temperature (chilling, freezing, and heat), salinity, drought, and heavy metal toxicity treatment. For instance, after being treated with different temperatures, the total endogenous PAs, Spd, Spm, and Put in the cabbage leaves exhibited an initial increase followed by a decrease: High-temperature treatment can promote an increase in the total PAs and Put in a short time, but as the duration of treatment extends, the synthesis of PAs is inhibited; moreover, low temperatures promote the synthesis of Spm, while high temperatures promote the synthesis of Spd [13]. Evidence indicates that the exogenous application of PAs can protect plants from damage caused by various abiotic stresses. Exogenous spray applications of Spd or Spm enhance the freezing tolerance of seedlings, including cucumber, tomato, and mung bean [14,15,16]. Spd or Spm also improve the heat tolerance of soybean seedlings [17]. Furthermore, Spm confers short-term salt tolerance to cucumber, lemon balm, and chrysanthemum [18,19,20]. Seed treatment with 0.1 mM putrescine effectively enhances the drought resistance of maize [21]. The exogenous application of Spd and Spm significantly promotes the germination of wheat seeds under drought stress [22]. Mung bean seeds treated with a combination of Put, Spd, and Spm demonstrate improved drought resistance [23].

Moreover, evidence has emerged suggesting that PAs play a role, either directly or indirectly, in modulating multiple pathways involved in programmed cell death (PCD) [24]. Overexpression of tomato SAMDC, driven by a maturity-specific promoter, significantly elevated Spd and Spm levels, inhibiting fruit senescence and extending shelf life [25]. In contrast to wild-type plants, downregulation of *samdc* in transgenic plants leads to a marked decrease in the levels of Spd and Spm, and under stressful conditions, these plants exhibit a heightened induction of PCD [26].

Previous studies have revealed the impact of exogenous PAs on enhancing plant tolerance to abiotic stresses, but their effect on freezing tolerance in rapeseed still remains largely unknown. Therefore, in this study, the influence of exogenous Spd on the freezing tolerance in three different cultivars of rapeseed with varying freezing sensitivity was evaluated. Additionally, through transcriptomic analysis, the possible mechanisms mediated by Spd in freezing tolerance were investigated.

## 2. Materials and Methods

### 2.1. Plant Materials

We chose the extremely early-maturing *B. napus* variety 862, the early-maturing rapeseed variety Westar, and the medium-maturing rapeseed variety ZS11 to evaluate the differences in freezing tolerance resulting from the exogenous application of Spd. The rapeseed seeds with consistent germination were picked out and moved to the plug trays (5 rows × 3 columns) filled with soil and vermiculite, and were grown to the four-leaf and one-heart stage under the conditions of 6:00–22:00/20 °C (light) and 22:00–6:00/16 °C (dark) for subsequent freezing treatment and phenotypic analysis. The humidity was maintained at 50%, and the light intensity was set to 10,000 lux.

### 2.2. Freezing Treatment

A total of ninety uniform 862 rapeseed seedlings with consistent growth were divided evenly into three groups. At 10:00, the seedlings in each group were sprayed with 30 mL of 0 mM Spd (ddH_2_O), 0.1 mM Spd, and 1.0 mM Spd solutions, respectively. Following the spraying, they were left to dry for 2 h at 20 °C. Afterwards, half of the seedlings from each concentration treatment (15 seedlings per group) were maintained at 20 °C as the control group, while the remaining 15 seedlings from each concentration treatment underwent a freezing treatment. This treatment involved gradually lowering the temperature from 20 °C to 4.0 °C between 12:00 and 12:30 (with a humidity of 75%), further decreasing from 4.0 °C to 0 °C between 12:30 and 13:00, and then reducing the temperature by 1.0 °C every hour from 13:00 to 17:00, reaching −4.0 °C (with a humidity of 75%). At −4.0 °C, three seedlings from each of the three treatments (both with and without freezing treatment) were quickly frozen in liquid nitrogen and then stored at −80 °C for subsequent H_2_O_2_ content measurement and RNA extraction. After sampling, the freezing treatment group continued to be treated at −4 °C for an additional 3.5 h and was gradually returned to 20 °C after 20:30. All of the aforementioned treatments were replicated in three biological replicates, with the same treatment protocol applied consistently to both Westar and ZS11.

Damage was categorized into the following five grades based on severity: Grade 0: No visible damage to the rapeseed; Grade 1: Damage with wilting of two or fewer fully expanded leaves, with the heart leaf remaining undamaged; Grade 2: Damage with wilting of three to four fully expanded leaves; Grade 3: Damage with wilting of four fully expanded leaves, with the heart leaf being normal or slightly damaged, and the plant capable of regaining growth; Grade 4: Severe damage to all leaves and the heart leaf, with the plant nearing death. The number of plants in each grade (N0 to N4) was recorded. The freezing injury index was calculated using the following formula: [(1 × N1) + (2 × N2) + (3 × N3) + (4 × N4)]/[4 × (N0 + N1 + N2 + N3 + N4)] × 100%.

### 2.3. Determination of H_2_O_2_ Concentration

H_2_O_2_ content was determined based on the titanium chloride colorimetric method, as previously described by Chakrabarty et al. [27]. The experiment was performed with three biological replicates, each having three technical replicates.

### 2.4. RNA-Seq

Utilizing the SteadyPure Plant RNA Extraction Kit (Accurate Biotechnology, Changsha, China), total RNA from rapeseed leaves was extracted according to the manufacturer’s instructions. The above-ground parts of Westar seedlings, treated with ddH_2_O (NSpd_C) and 1.0 mM Spd (HSpd_C) under normal conditions and with ddH_2_O (NSpd_F) and 1.0 mM Spd (HSpd_F) under freezing stress conditions, were rapidly frozen in liquid nitrogen. Each treatment required two biological replicates. After RNA extraction from the tissues, cDNA libraries were constructed and subjected to transcriptome sequencing. The data quality control is presented in Table S2.

### 2.5. Quantitative Real-Time PCR Analysis

The first strand of cDNA was synthesized using the FastKing Reverse Transcription Kit, and then the resulting cDNA was diluted 100-fold for subsequent use in RT-PCR reactions. The ChamQ Universal SYBR qPCR Kit was employed for the RT-qPCR analysis, conducted on a Gentier 48E Real-time PCR system. *BnaActin* (*BnaC05g34300D*) was used as the reference gene, and the relative expression levels were calculated using the 2^−ΔCt^ method. The RT-qPCR primers and their sequences are shown in Appendix A Table A1.

### 2.6. Statistics and Analysis

We analyzed the data through a one-way ANOVA using SPSS 25, and plotted the data using GraphPad Prism (version 9.0.0) and TBtools (version 2.144) [28].

## 3. Results

### 3.1. Two Concentrations of Exogenous Spd Improve the Freezing Tolerance of Seedlings

**Figure 1:**
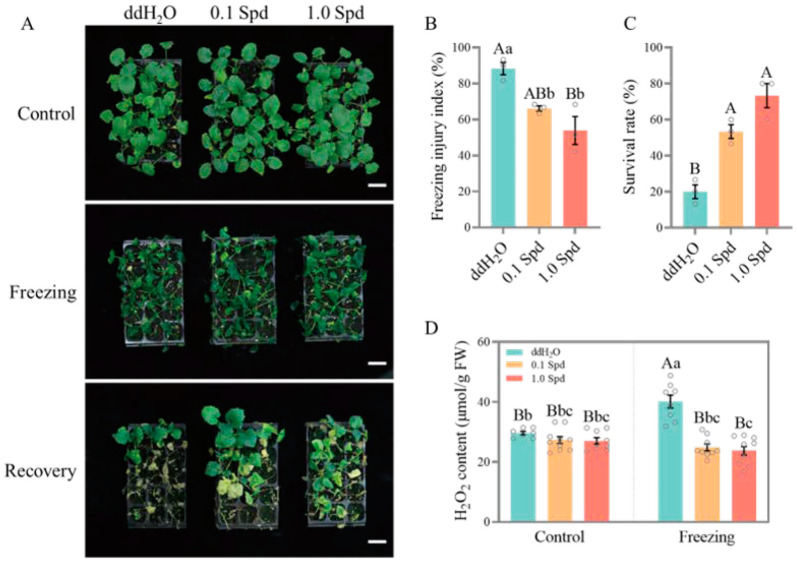
The exogenous Spd at concentrations of 0.1 mM and 1 mM significantly enhanced the freezing tolerance of extremely early-maturing variety 862. (**A**) Photographs of 862 seedlings sprayed with ddH_2_O, 0.1 mM Spd, and 1.0 mM Spd before and after freezing treatment, and after recovery for 7 days, bar = 5 cm; (**B**,**C**) freezing injury index (**B**) and survival rate (**C**) of seedlings sprayed with ddH_2_O, 0.1 mM Spd, and 1.0 mM Spd after freezing treatment; (**D**) H_2_O_2_ content in seedlings sprayed with ddH_2_O, 0.1 mM Spd, and 1.0 mM Spd under normal conditions and freezing treatment. Values represent mean ± SE. (**B**,**C**): A one-way ANOVA was conducted with *n* = 3, encompassing three biological replicates, each containing 15 individual plants. (**D**): A two-way ANOVA was performed with *n* = 9, which comprised three biological replicates and three technical replicates. In both sets of analyses, a post hoc test using the Tukey’s HSD test was applied. Within these analyses, different lowercase letters indicate statistically significant differences (*p* < 0.05), while a combination of different lowercase and uppercase letters denotes extremely significant differences (*p* < 0.01).

**Figure 2:**
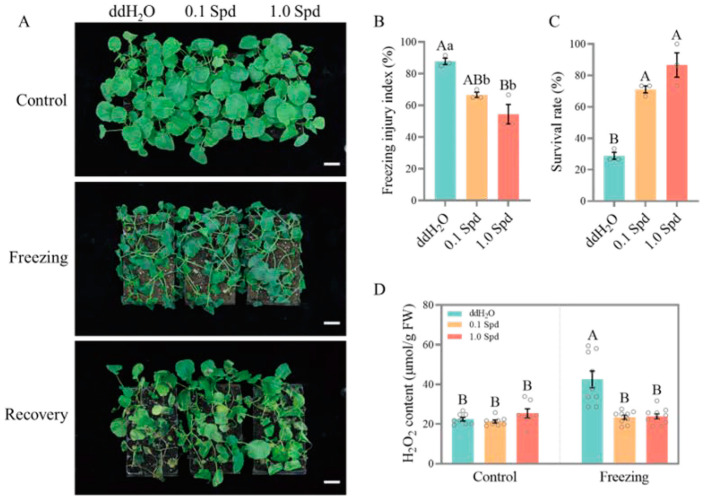
The exogenous Spd at concentrations of 0.1 mM and 1 mM significantly enhanced the freezing tolerance of early-maturing variety Westar. (**A**) Photographs of Westar seedlings sprayed with ddH_2_O, 0.1 mM Spd, and 1.0 mM Spd before and after freezing treatment, and after recovery for 7 days, bar = 5 cm; (**B**,**C**) freezing injury index (**B**) and survival rate (**C**) of seedlings sprayed with ddH_2_O, 0.1 mM Spd, and 1.0 mM Spd after freezing treatment; (**D**) H_2_O_2_ content in seedlings sprayed with ddH_2_O, 0.1 mM Spd, and 1.0 mM Spd under normal conditions and freezing treatment. Values represent mean ± SE. (**B**,**C**): A one-way ANOVA was conducted with *n* = 3, encompassing three biological replicates, each containing 15 individual plants. (**D**): A two-way ANOVA was performed with *n* = 9, which comprised three biological replicates and three technical replicates. In both sets of analyses, a post hoc test using the Tukey’s HSD test was applied. Within these analyses, different lowercase letters indicate statistically significant differences (*p* < 0.05), while a combination of different lowercase and uppercase letters denotes extremely significant differences (*p* < 0.01).

**Figure 3:**
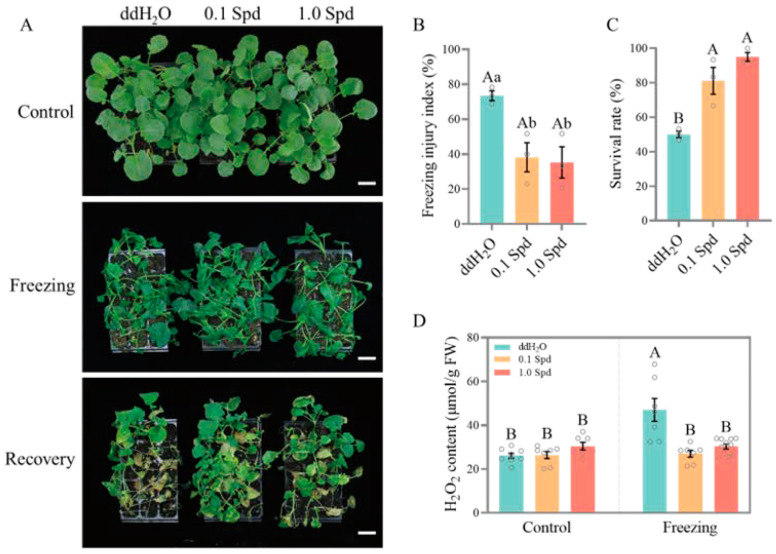
The exogenous Spd at concentrations of 0.1 mM and 1 mM significantly enhanced the freezing tolerance of extremely mid-maturing variety ZS11. (**A**) Photographs of ZS11 seedlings sprayed with ddH_2_O, 0.1 mM Spd, and 1.0 mM Spd before and after freezing treatment, and after recovery for 7 days, bar = 5 cm; (**B**,**C**) Freezing injury index (**B**) and Survival rate (**C**) of seedlings sprayed with ddH_2_O, 0.1 mM Spd, and 1.0 mM Spd after freezing treatment; (**D**) H_2_O_2_ content in seedlings sprayed with ddH_2_O, 0.1 mM Spd, and 1.0 mM Spd under normal conditions and freezing treatment. Values represent mean ± SE. (**B**,**C**): A one-way ANOVA was conducted with *n* = 3, encompassing three biological replicates, each containing 15 individual plants. (**D**): A two-way ANOVA was performed with *n* = 9, which comprised three biological replicates and three technical replicates. In both sets of analyses, a post hoc test using the Tukey’s HSD test was applied. Within these analyses, different lowercase letters indicate statistically significant differences (*p* < 0.05), while a combination of different lowercase and uppercase letters denotes extremely significant differences (*p* < 0.01).

In order to evaluate the role of exogenous Spd in the freezing tolerance of rapeseed, we selected three rapeseed genotypes: 862 (an extremely early-maturing variety), Westar (a spring-type cultivar), and ZS11 (a semi-winter-type cultivar), and carried out freezing treatment on the seedlings sprayed with different concentrations (0, 0.1, and 1.0 mM) of Spd. Results showed that ZS11 had the strongest freezing tolerance (50% survival rate), Westar had a moderate tolerance (28.89% survival rate), and 862 had the weakest (20% survival rate) (Figure 1, Figure 2 and Figure 3). However, exogenous Spd (both 0.1 mM and 1.0 mM) application significantly improved the freezing tolerance of 862, Westar, and ZS11 seedlings compared to 0 mM Spd (ddH_2_O), and the effect of 1.0 mM Spd is better than 0.1 mM Spd (Figure 1, Figure 2 and Figure 3). For 862 seedlings, after spraying with 0.1 mM and 1.0 mM Spd, the freezing injury index reduced from 88.33% to 66.11% and 53.89%, respectively, while the survival rates increased from 20% to 53.33% and 73.33%, respectively. H_2_O_2_ accumulation levels in rapeseed seedlings were also measured to further evaluate the role of Spd under freezing treatment. As shown in Figure 1, Figure 2 and Figure 3, spraying Spd significantly reduced the accumulation level of H_2_O_2_ in the rapeseed seedlings under freezing treatment, while no significant differences were observed under normal growth conditions. The results indicate that exogenous application of Spd at concentrations of 0.1 mM and 1.0 mM enhances the freezing tolerance of rapeseed seedlings by reducing the production of the reactive oxygen species H_2_O_2_.

### 3.2. Transcriptome Sequencing Analysis of the Effect of Spd on Rapeseed Seedlings under Freezing Treatment

To further reveal the underlying regulatory mechanism of Spd in the freezing response of rapeseed seedlings, we employed transcriptome sequencing analysis to explore the differentially expressed genes (DEGs) in the above-ground parts of Westar seedlings treated with or without freezing stress after being sprayed with ddH_2_O and 1.0 mM Spd. A set of four libraries was constructed, and a specified amount of 5.08~7.20 GB of clean reads was obtained (Table S5).

**Figure A1:**
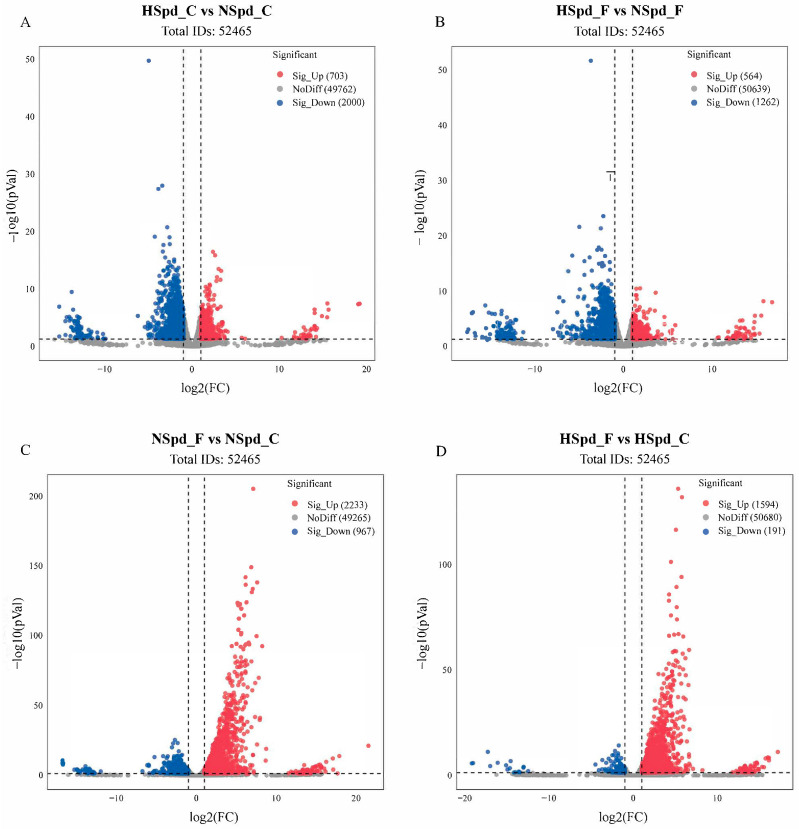
Volcano plots of differentially expressed genes (DEGs) under normal and freezing treatment conditions after spraying with ddH_2_O or 1.0 mM Spd. (**A**) HSpd_C vs. NSpd_C volcano plot; (**B**) HSpd_F vs. NSpd_F volcano plot; (**C**) NSpd_F vs. NSpd_C volcano plot; (**D**) HSpd_F vs. HSpd_C volcano plot.

Initially, we compared the differentially expressed genes (DEGs) between the samples with ddH_2_O and 1.0 mM Spd (HSpd_C vs. NSpd_C) under normal circumstances, identifying 2703 DEGs, with 703 up-regulated and 2000 down-regulated genes. This suggests that the expression of these DEGs is influenced by exogenous Spd. Notably, the number of down-regulated genes significantly exceeds that of the up-regulated genes, indicating that Spd has a stronger inhibitory effect on gene expression compared to its activation effect. Additionally, we compared the differential genes (HSpd_F vs. NSpd_F) under freezing stress treatment with the application of ddH_2_O and 1.0 mM Spd, identifying 1826 DEGs, including 564 up-regulated and 1262 down-regulated genes, which may contribute to the differences in freezing tolerance between treatments with ddH_2_O and 1.0 mM Spd. Subsequently, we also compared the differential genes under freezing stress and normal conditions for the application of ddH_2_O or 1.0 mM Spd. Under freezing treatment conditions, we identified 4966 DEGs in the samples treated with ddH_2_O (NSpd_F vs. NSpd_C), with 3013 up-regulated and 1953 down-regulated, indicating that freezing stress activated more gene expressions, as evidenced by a higher number of up-regulated genes compared to down-regulated ones. Similarly, in the samples sprayed with 1.0 mM Spd under freezing treatment conditions (HSpd_F vs. HSpd_C), a total of 3014 DEGs were identified, among which 2325 genes were up-regulated in expression and 689 genes were down-regulated in expression, and the number of up-regulated genes was significantly higher than that of down-regulated genes. The number of DEGs identified by HSpd_F vs. HSpd_C is much lower than that identified by NSpd_F vs. NSpd_C, suggesting the important regulatory role of Spd in the response to freezing damage stress (Appendix B Figure A1).

**Figure 4:**
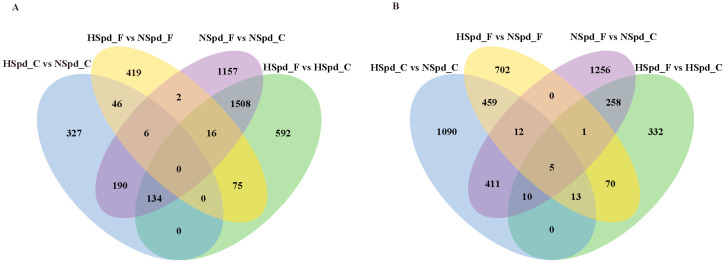
Venn diagram analysis of differential gene expression (DEGs) profiles in early-maturing variety Westar after treatment with different concentrations of Spd (0 mM vs. 1 mM) under normal and freezing treatment. (**A**) The overlapping up-regulated differentially expressed genes (DEGs) across the four comparative pairs: HSpd_C vs. NSpd_C, HSpd_F vs. HSpd_C, NSpd_F vs. NSpd_C, and HSpd_F vs. NSpd_F. (**B**) The overlapping down-regulated DEGs across the four comparative pairs: HSpd_C vs. NSpd_C, HSpd_F vs. HSpd_C, NSpd_F vs. NSpd_C, and HSpd_F vs. NSpd_F. HSpd_C: 1 mM Spd, 20 °C; NSpd_C: ddH_2_O, 20 °C; HSpd_F: 1 mM Spd, −4 °C; NSpd_F: ddH_2_O, −4 °C.

Overlap between the identified DEGs from the comparison groups was conducted through Venn diagrams to further analyze the roles of these DEGs (Figure 4). The results revealed a total of 1957 overlapping genes between HSpd_F vs. HSpd_C and NSpd_F vs. NSpd_C, indicating a conservation in the regulation of these genes under freeze stress, regardless of whether ddH_2_O or 1.0 mM Spd treatments. Intersection of these 1957 DEGs with the comparison group HSpd_F vs. NSpd_F identified 82 genes showing differential expression, suggesting a potential association of these genes with the differences in freezing tolerance between the ddH_2_O and 1.0 mM Spd treatments. Similarly, by overlapping the 1957 freezing stress-induced differentially expressed genes (DEGs) with the comparison of HSpd_C vs. NSpd_C, 222 genes were found to be differentially expressed in the HSpd_C vs. NSpd_C comparison, indicating that these Spd-induced genes are involved in the response to freezing stress (Figure 4).

### 3.3. Gene Ontology Functional and Kyoto Encyclopedia of Genes and Genomes Pathway Enrichment Analysis

**Figure 5:**
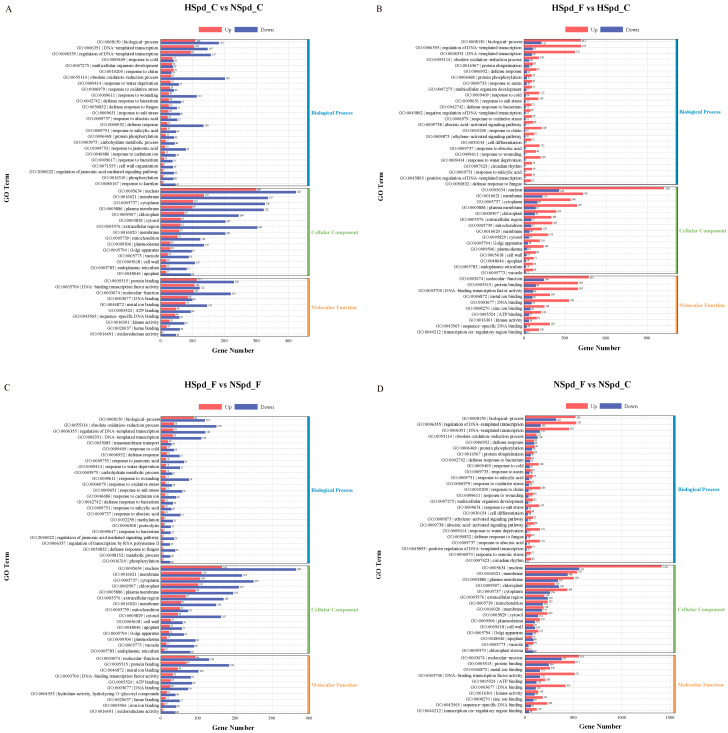
Gene ontology (GO) enrichment analysis of DEGs under normal and freezing treatment conditions after spraying with ddH_2_O or 1.0 mM Spd. (**A**) HSpd_C vs. NSpd_C GO enrichment analysis; (**B**) HSpd_F vs. HSpd_C GO enrichment analysis; (**C**) HSpd_F vs. NSpd_F GO enrichment analysis; (**D**) NSpd_F vs. NSpd_C GO enrichment analysis.

**Figure 6:**
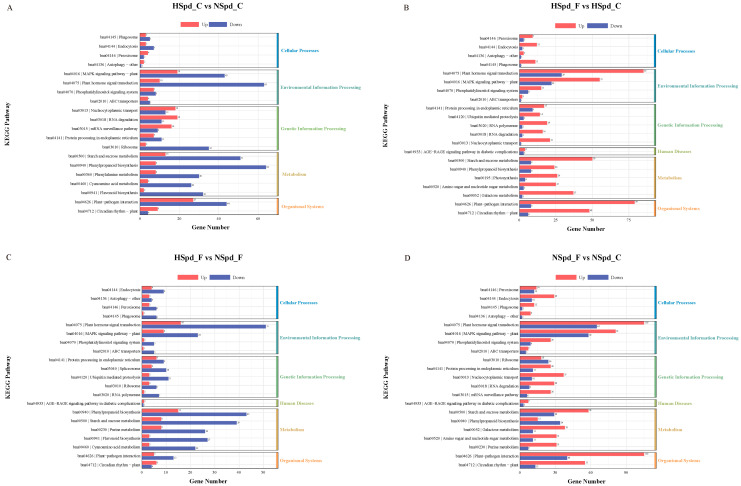
Kyoto Encyclopedia of Genes and Genomes (KEGG) enrichment analysis of DEGs under normal and freezing treatment conditions after spraying with ddH_2_O or 1.0 mM Spd. (**A**) HSpd_C vs. NSpd_C KEGG enrichment analysis; (**B**) HSpd_F vs. HSpd_C KEGG enrichment analysis; (**C**) HSpd_F vs. NSpd_F KEGG enrichmen analysis t; (**D**) NSpd_F vs. NSpd_C KEGG enrichment analysis.

First, we conducted a GO functional enrichment analysis for each comparative group, presenting the top 35 significantly enriched GO terms (Figure 5). Subsequently, to further elucidate biological functions, we performed a KEGG pathway enrichment analysis, focusing on the top five enriched pathways in the first-level classification of KEGG (Figure 6).

**Figure 7:**
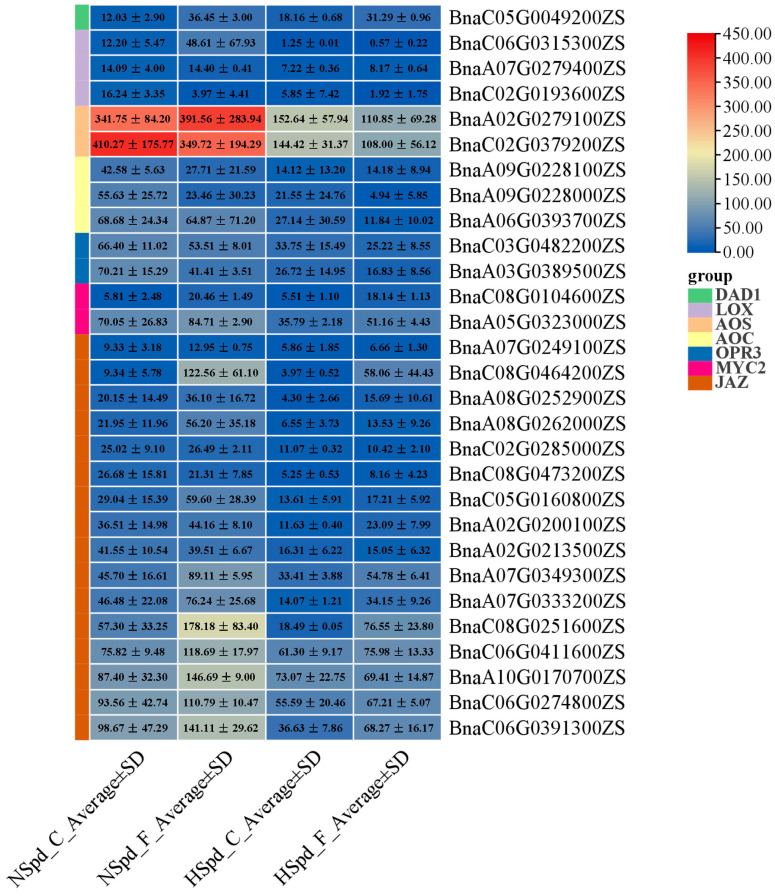
Expression changes of genes related to the jasmonic acid (JA) pathway in response to normal and freezing treatments after spraying with ddH_2_O or 1.0 mM Spd.

Our KEGG pathway enrichment analysis of DEGs across the four comparative groups revealed that the “plant hormone signal transduction” pathway was consistently present, regardless of whether ddH_2_O or Spd were applied, with or without freezing stress treatment. This finding underlines the pathway’s importance in the polyamine-mediated variance in freezing tolerance. Additionally, the GO term “response to jasmonic acid” and terms related to oxidation–reduction processes were prevalent across all groups. Consequently, we utilized cluster analysis to study the expression changes of genes associated with the jasmonic acid signaling pathway (Figure 7).

Spd may indirectly modulate the response to freezing stress via the jasmonic acid signaling pathway. Typically, under normal conditions, this pathway may be suppressed by Spd. In comparisons such as HSpd_F vs. HSpd_C and NSpd_F vs. NSpd_C, the upregulation of genes in most categories surpassed that of downregulation, implying that freezing stress predominantly induces gene expression. Conversely, in the GO enrichment analysis of HSpd_C vs. NSpd_C and HSpd_F vs. NSpd_F, the number of downregulated genes generally exceeded that of upregulated ones, suggesting that Spd application predominantly inhibits gene expression. Although this inhibitory effect is somewhat mitigated under freezing stress, the suppression remains markedly greater than any promotion. Notably, among the JA signaling-related genes, *LOX* (*BnaC06G0315300ZS*), which is differentially expressed across all comparative groups, is induced by both freezing stress and Spd. This gene is a pivotal element in the JA signal transduction pathway, highlighting its crucial role in the freezing tolerance conferred by Spd (Figure 7).

**Figure 8:**
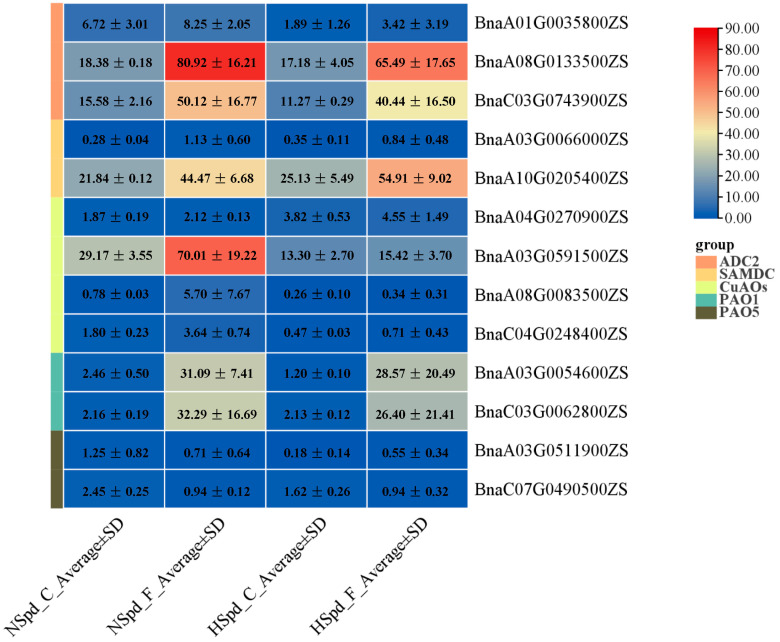
Expression changes of genes related to polyamines (PAs) metabolism pathway under normal and freezing treatments after spraying with ddH_2_O or 1.0 mM Spd.

To further investigate the Spd-mediated response mechanism to freezing stress, the DEGs involved in the PA biosynthesis and metabolism were analyzed. Unexpectedly, the majority of genes involved in the PA biosynthesis and metabolic pathway do not respond to the exogenous application of Spd or freezing treatment, only fourteen genes (six PA biosynthesis-related genes and eight PA oxidative catabolism-related genes) responded to Spd application or freezing treatment (Figure 8). All six PA synthesis-related genes were induced by freezing stress and showed different degrees of up-regulation in their expression, especially two *ADC2* genes (*BnaA08G0133500ZS*, *BnaC03G0743900ZS*) and one *SAMDC* gene (*BnaA10G0205400ZS*), indicating that freezing stress may promote the synthesis and accumulation of plant PAs. Under normal conditions, it appears that only the expression of one *ADC2* gene (*BnaA01G0035800ZS*) is inhibited by Spd, while other polyamine biosynthesis-related genes show no significant changes. Under freezing stress conditions, except for one *SAMDC* gene (*BnaA10G0205400ZS*), Spd seems to slightly suppress the overall expression of polyamine biosynthesis-related genes, implying that exogenous Spd application plays a role in maintaining polyamine biosynthesis under freezing stress. Similarly, freezing stress induces the expression of polyamine oxidase-related genes, indicating that activated amine oxidases play a key role in maintaining polyamine levels and in responding to freezing stress.

**Figure 9:**
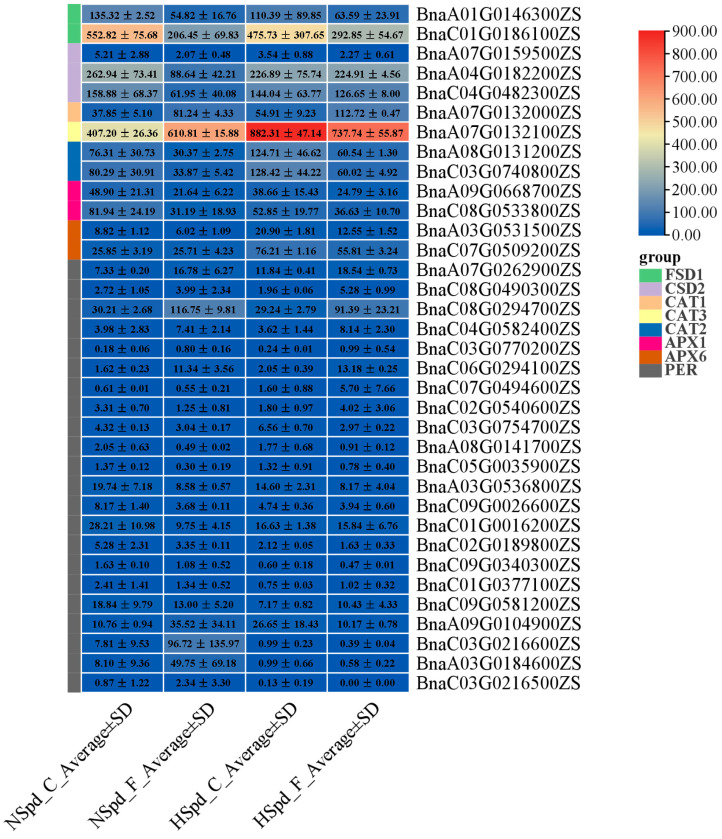
Expression changes of genes related to the antioxidant system under normal and freezing treatments after spraying with ddH_2_O or 1.0 mM Spd.

Additionally, we analyzed the expression patterns of the antioxidant system (mainly antioxidant enzymes, including genes related to *SOD*, *POD*, *CAT*, and *APX*) among different samples (Figure 9). Among the thirteen genes related to *SOD*, *CAT*, and *APX*, the exogenous application of Spd strongly induced the expression of four *CAT*-related genes (*BnaA07G0132000ZS*, *BnaA07G0132100ZS*, *BnaA08G0131200ZS*, *BnaC03G0740800ZS*) and two *APX6*-related genes (*BnaA03G0531500ZS*, *BnaC07G0509200ZS*), while slightly repressing the expression of other genes; however, their overall expression levels remained high. Under freezing stress treatment, the expression levels of other genes were inhibited, except for *CAT1* and *CAT3*, and this inhibitory effect seemed to be alleviated after the application of Spd, as evidenced by higher levels of expression in HSpd_F compared to NSpd_F, suggesting that the enhanced antioxidant mechanism may be one of the reasons why Spd improves the freezing tolerance of *B. napus*.

Among the 29 *PERs*, most genes showed a low-abundance of expressions in various samples. Therefore, we focused on nine *PERs* with high-level expressions (*BnaA07G0262900ZS*, *BnaC08G0294700ZS*, *BnaC06G0294100ZS*, *BnaA03G0536800ZS*, *BnaC01G0016200ZS*, *BnaC09G0581200ZS*, *BnaA09G0104900ZS*, *BnaC03G0216600ZS*, and *BnaA03G0184600ZS*) and discovered different expression patterns from genes related to other antioxidant enzymes. Exogenous Spd inhibited the expression of most *PER* genes and only increased the expression level of some *PERs* to a certain extent, resulting in a reduction in their overall expression level. Except for three *PERs* (*BnaA03G0536800ZS*, *BnaC01G0016200ZS*, and *BnaC09G0581200ZS*), freezing stress strongly induced the expression levels of most *PERs*, and the up-regulation of this expression level was only significantly inhibited by Spd in a small portion, while the expression levels of most other genes did not change significantly (Figure 9).

### 3.4. qRT-PCR Validation of DEGs

**Figure 10:**
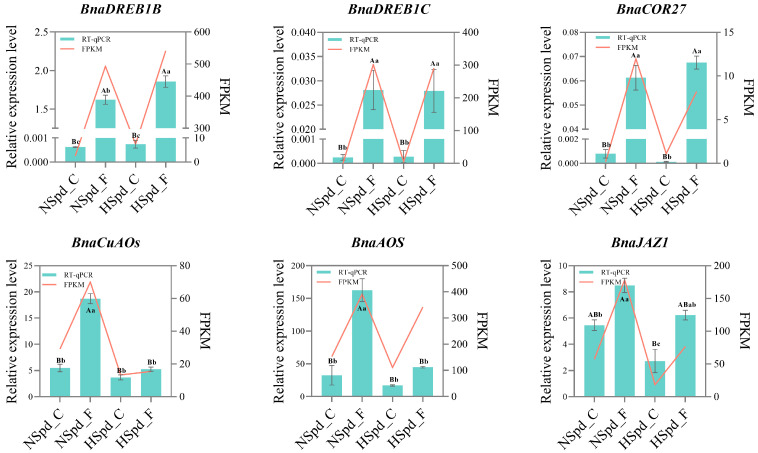
qRT-PCR validation of gene expression under normal and freezing treatments after spraying with ddH_2_O or 1.0 mM Spd. The genes order from left to right and top to bottom are *BnaDREB1B*, *BnaDREB1C*, *BnaCOR27*, *BnaCuAOs*, *BnaAOS*, and *BnaJAZ1*. The red line graph represents the gene’s FPKM values under normal and freezing treatments after spraying with ddH_2_O or 1.0 mM Spd, while the green bar chart illustrates the qRT-PCR validation of gene expression under normal and freezing treatments after spraying with ddH_2_O or 1.0 mM Spd. The experimental data are presented as mean ± standard error (SE), and a one-way ANOVA was performed with *n* = 3, including three technical replicates. In both sets of analyses, a post hoc test using the Tukey’s HSD test was applied. In these analyses, different lowercase letters signify statistically significant differences (*p* < 0.05), whereas a combination of different lowercase and uppercase letters indicates extremely significant differences (*p* < 0.01).

**Figure A2:**
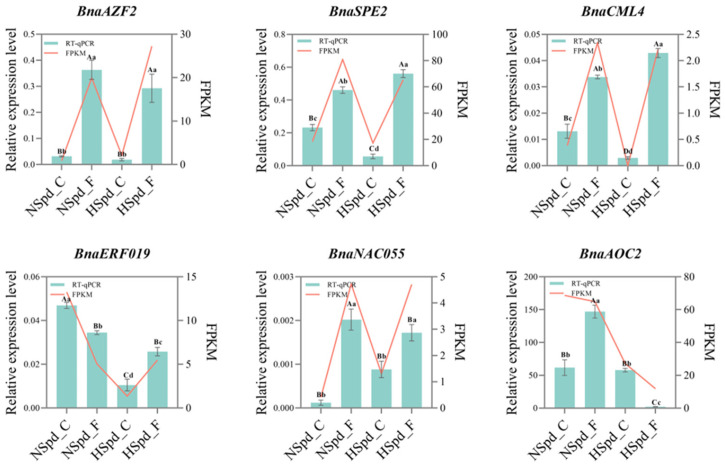
qRT-PCR validation of gene expression under normal and freezing treatments after spraying with ddH_2_O or 1.0 mM Spd. The genes order from left to right and top to bottom are *BnaAZF2*, *BnaSPE2*, *BnaCML4*, *BnaERF019*, *BnaNAC055*, and *BnaAOC2*. The red line graph represents the gene’s FPKM values under normal and freezing treatments after spraying with ddH_2_O or 1.0 mM Spd, while the green bar chart illustrates the qRT-PCR validation of gene expression under normal and freezing treatments after spraying with ddH_2_O or 1.0 mM Spd. The experimental data are presented as mean ± standard error (SE), and a one-way ANOVA was performed with *n* = 3, including three technical replicates. In both sets of analyses, a post hoc test using the Tukey’s HSD test was applied. In these analyses, different lowercase letters signify statistically significant differences (*p* < 0.05), whereas a combination of different lowercase and uppercase letters indicates extremely significant differences (*p* < 0.01).

To validate the reliability of the transcriptome data, we randomly selected several genes from the DEGs for qRT-PCR validation, including freezing stress response genes, such as *BnaDREB1C* (*BnaA03G0486800ZS*), *BnaDREB1B* (*BnaC08G0165900ZS*), and *BnaCOR27* (*BnaA06G0444700ZS*), as well as a polyamine oxidases gene, *BnaCuAOs (BnaA03G0591500ZS),* and JA-signaling genes, *BnaAOS* (*BnaA02G0279100ZS*) and *BnaJAZ1* (*BnaC08G0251600ZS*). The results showed that the relative expression levels of these genes across different samples were generally consistent with the overall trend of the transcriptome data, suggesting a high reliability of the transcriptome data (Figure 10). Gene expression profiles for conditions of normal temperature and freezing stress are presented in Appendix B Figure A2.

## 4. Discussion

A large number of research studies of various plant species have confirmed that PA accumulation occurs in response to several adverse environmental conditions, including extreme temperature (chilling, cold, and heat), salinity, drought, and heavy metal toxicity treatment [6]. In this study, freezing stress significantly induced the expression level of genes involved in PAs biosynthesis (*ADC2* and *SAMDC*) and PAs metabolism (*CuAOs* and *PAO1*) in rapeseed seedlings (Figure 8), which indicated that the PA pathway is stimulated by freezing stress and plays an important role in the adaptability of rapeseed seedlings to freezing stress. We further showed that the exogenous application of two concentrations of Spd significantly improved the freezing tolerance of three different maturity rapeseed types, as demonstrated by higher survival rates and lower freezing injury indices (Figure 1, Figure 2 and Figure 3). Although there is still controversy about the molecular mechanism by which polyamines improve the tolerance of plants to abiotic stresses at present, it is undeniable that polyamines play a key role in the homeostasis of the ROS. As shown in Figure 1, Figure 2 and Figure 3, under freezing treatment, the freezing-induced H_2_O_2_ accumulation was significantly impaired after the application of Spd in rapeseed seedlings. This may be caused by the repression of CuAOs and PAOs enzymes. These results indicated that freezing stress can induce CuAOs and PAOs activities, resulting in a large accumulation of H_2_O_2_, which causes oxidative damage in rapeseed seedlings. While exogenous application of Spd reduced the freezing-induced CuAOs/PAOs activities and H_2_O_2_ content in rapeseed seedlings by down-regulating the expression level of *CuAOs* and *PAOs*. Simultaneously, under freezing treatment, exogenous application of Spd had a higher expression level of antioxidant enzyme-related genes than ddH_2_O (HSpd_F vs. NSpd_F), providing transcriptional-level support for this possibility. The application of Spd may endow plants with enhanced antioxidant mechanisms, conferring a stronger capability to mitigate the damage caused by hydrogen peroxide. This aligns with the findings of previous research [29,30].

The application of exogenous Spd enhances the freezing tolerance of different maturity-stage varieties of *B. napus* L. by reducing H_2_O_2_. Combined with transcriptome analysis, we propose the following hypothesis for the working model of how Spd improves the freezing tolerance of *B. napus*: Freezing stress induces the expression of *ADC2* and *SAMDC*, leading to the biosynthesis and accumulation of Put and Spd. This, in turn, activates the activity of CuAOs and PAOs, which catalyze the oxidative degradation of polyamines. When plants are subjected to severe freezing stress, the balance cannot be maintained, leading to an ROS burst, which causes cell damage and even plant death. After spraying with an appropriate concentration of Spd followed by freezing treatment, the expression of CuAOs was downregulated, which significantly reduced the synthesis of hydrogen peroxide. This prevented H_2_O_2_ bursts, avoided cellular damage, and consequently enhanced the freezing tolerance of rapeseed seedlings. Furthermore, the interactions between polyamines and other related pathways, such as plant hormone signaling pathways and MAPK signaling pathways, also played an indispensable role (Figure 9). The synergistic effect of PAs and specific hormones (IAA, CTK, and GA3) promotes the germination of cotton seeds under cold stress, while ABA plays an opposite role [31]. Exogenous Spm enhances the cold tolerance of sweet corn seedlings by modulating ABA levels, ROS homeostasis, and calcium ion transport pathways [32].

The abiotic stress-signaling network is highly intricate. In fact, plant hormones regulate the expression of PA biosynthesis and metabolic genes, and PAs can regulate the biosynthesis and signal transduction of plant hormones [33,34]. In the *adc1* and *adc2* mutants, decreased expression levels of the key ABA biosynthesis gene *NCED3* were observed, and complementation experiments with ABA indicated that Put regulates ABA levels in response to cold acclimation and freezing tolerance in *Arabidopsis* [35]. Spd boosts the expression of *GT-3b*, a trihelix transcription factor that responds to light and stress in plants [36]. Additionally, under salt stress conditions, Spd also increases GA biosynthesis, enhances the activity of related oxidases, and promotes the accumulation of endogenous GA levels [37]. Moreover, under salt stress, Spd activates the expression of RBOH (respiratory burst oxidase homolog), which also plays a pivotal role in plant responses to wounding and JA [38,39]. In this study, we found that the “Plant Hormone Signal Transduction” pathway was significantly enriched in all four comparison groups. Moreover, responses to plant hormones such as JA, ABA, SA, and ethylene were assigned to each comparison group. Notably, the GO term “response to jasmonic acid” was discovered to be concurrently enriched in the GO functional annotations of all four comparison groups. One key gene in the JA pathway, *MYC2* (*BnaA05G0323000ZS*), is induced by Spd and freezing stress, indicating that Spd can participate in the JA signaling pathway through transcriptional regulation to modulate the plant’s JA levels and its response to freezing stress. Polyamines can cross-regulate plant responses to stress with JA [40]. Research shows that sugar beet plants treated with methyl jasmonate (MeJA) exhibit enhanced resistance to the beet mosaic virus (BtMV), which is closely related to the increased accumulation of PAs and salicylic acid (SA) [41]. Spraying MeJA on the leaves of wheat plants increases the levels of endogenous free and conjugated forms of Put, Spd, and Spm, thereby enhancing resistance to the development of wheat leaf rust disease [42]. Spm deficiency disrupts the balance of *Arabidopsis* defense responses, enhancing those regulated by JA relative to those by SA [43]. JA not only links polyamines to influence plant defense but also affects plant organogenesis. Previous studies have indicated that MeJA upregulates the expression of biosynthetic genes, promotes the oxidation and conjugation of PAs, and inhibits shoot formation in tobacco leaves [44]. However, the specific mechanisms by which PAs and the JA signaling pathway intersect to influence plant freezing tolerance remain unclear and necessitate further research.

## 5. Conclusions

This paper studied the impact of exogenous spraying of polyamines on *B. napus* seedlings. The survival rates of different maturity types of *B. napus* seedlings that were frozen after exogenous spraying with 0.1 Mm and 1 mM of Spd were significantly higher than those of rapeseed seedlings sprayed with ddH_2_O. Further research indicated that Spd regulated the freezing tolerance of *B. napus* by adjusting genes related to the PAs metabolic pathway and antioxidant mechanisms and also inhibited the expression of genes related to the JA signaling pathway through transcriptional regulation. To sum up, polyamines enhanced the freezing tolerance of *B. napus* seedlings by regulating reactive oxygen species. In subsequent work, we will continue to focus on the genes related to the PAs metabolic pathway that are simultaneously induced by Spd and freeze injury in order to better understand the freezing tolerance mechanism of *B. napus* and provide a direction for cultivating early-maturing and freezing-tolerant *B. napus* varieties.

## Data Availability

The data presented in this study are available upon request from the corresponding author. We have uploaded the transcriptome data to the NCBI database (BioProject: PRJNA1148091).

## Supplementary Materials

The following supporting information can be downloaded at: https://www.mdpi.com/article/10.3390/antiox13091032/s1, Table S1. Preparation table for H_2_O_2_ standard curve. Table S2: Sample quality control statistics and reference genome alignment read statistics table. Table S3: Expression changes of genes related to the jasmonic acid (JA) pathway in response to normal and freezing treatments after spraying with ddH_2_O or 1.0 mM Spd. Table S4: Expression changes of genes related to polyamines (PAs) metabolism pathway under normal and freezing treatments after spraying with ddH_2_O or 1.0 mM Spd. Table S5: Expression changes of genes related to the antioxidant system under normal and freezing treatments after spraying with ddH_2_O or 1.0 mM Spd.

## Appendix A

**Table A1:** Primer sequences.

Primer Name	ZS11 Gene ID	Primer Sequence (5′→3′)
	Darmor Gene ID	
Actin-F	*-*	GGTTGGGATGGACCAGAAGG
Actin-R	*BnaC05g34300D*	TCAGGAGCAATACGGAGC
DREB1C_qF	*BnaA03G0486800ZS*	CGGCAAATCAGCTTGTCTCAA
DREB1C_qR	*-*	GCAGCCGCCTTCTGAAATCT
AZF2_qF	*BnaA05G0364000ZS*	CAAGACGCAACCGCAAGAAT
AZF2_qR	*BnaA05g20490D*	GTTGTTGAATCGTCGTCGGC
COR27_qF	*BnaA06G0444700ZS*	GCCAAAACGGAAGCTCCAAG
COR27_qR	*BnaA06g37010D*	AGTGGAACAACCTATACGCCA
SPE2_qF	*BnaA08G0133500ZS*	CCGTATCTAGCGACTGAGCC
SPE2_qR	*BnaA08g10990D*	CCACACGACTACCCTTGTCA
CML4_qF	*BnaA09G0541500ZS*	ACCGACGACAAGATCACACC
CML4_qR	*BnaA09g37780D*	GCCGTCTCCGTTCTTGTCA
ERF019_qF	*BnaC05G0198700ZS*	TAGTTTCCAAGACCTCGCCG
ERF019_qR	*BnaC05g18050D*	CGTGATCGTGACCGTCCAAA
NAC055_qF	*BnaC05G0447000ZS*	TACGTTACGTTAACCGGGTCG
NAC055_qR	*BnaC05g38150D*	TACTTCCTGTCCCTTGGGCT
DREB1B_qF	*BnaC08G0165900ZS*	GAGTCTCCGGTAGGTGGTGA
DREB1B_qR	*-*	GTGACGTGTCTCCCGAAACT
JAZ1_qF	*BnaC08G0251600ZS*	CATCGCTCCTACCTCGAACC
JAZ1_qR	*BnaC08g18640D*	TGAGATAGATTACCGCATGCCA
AOS-qF	*BnaA02G0279100ZS*	TTCTCTTCACGCTCGGCTTCAC
AOS-qR	*BnaA02g23180D*	ACGGCTAGATCAGGAGGAGTCTC
AOC2-qF	*BnaA06G0393700ZS*	TTACAGCTTCTACTTCGGCGACTAC
AOC2-qR	*BnaA06g33410D*	GGTGATGGCGAGGAACGAGTC
CuAOs_qF	*BnaA03G0591500ZS*	CTTGATTCGGGTCGGGTCGTTTC
CuAOs_qR	*-*	GCGATACCTGAGAAGCCACGATG
